# The quality-of-life impacts and economic burden of X-linked retinitis pigmentosa caused by variants in *RPGR*

**DOI:** 10.1038/s41433-026-04532-y

**Published:** 2026-05-21

**Authors:** Deborah Schofield, Joshua Kraindler, Rupendra N. Shrestha, Owen Tan, Sarah West, Natalie Hart, Lauren Wall, Liny Tan, Dustin Hewett, Lorraine Villaret, Alan Ma, John R. Grigg, Robyn V. Jamieson

**Affiliations:** 1 GenIMPACT: Centre for Economic Impacts of Genomic Medicine, Macquarie Business School, Macquarie University, Sydney, NSW Australia; 2 Eye Genetics Research Unit, Sydney Children’s Hospitals Network, Children’s Medical Research Institute, Save Sight Institute, University of Sydney, Sydney, NSW Australia; 3 Specialty of Genomic Medicine, University of Sydney, Sydney, NSW Australia; 4 Specialty of Ophthalmology, The University of Sydney, Sydney, NSW Australia

**Keywords:** Retinal diseases, Health care economics

## Abstract

**Objectives:**

To estimate the health and societal costs and health-related quality of life (HRQoL) impacts of X-linked retinitis pigmentosa (XLRP) caused by *RPGR*.

**Methods:**

Primary data were obtained from a clinical cohort of 9 patients with X-linked retinitis pigmentosa (XLRP) and 4 caregivers. Health-related quality of life (HRQoL) impacts for patients and carers are assessed using utility values derived from the Assessment of Quality of Life–8 Dimension (AQoL-8D) instrument. Lifetime costs associated with XLRP are estimated through microsimulation modelling. The model accounts for both societal and healthcare costs, capturing the economic burden on patients, caregivers and their spouses.

**Results:**

Average AQoL-8D utility values for patients with XLRP are 0.57, slightly lower than other inherited retinal diseases and significantly (*p* < 0.05) below age and gender adjusted population norms of 0.81. HUI2 and HUI3 values were 0.81 and 0.75, respectively. Lifetime costs for XLRP were AUD 7.0 m. Most costs (79%) were societal costs. Using a range of prevalence estimates, national annual costs are estimated to be between AUD 38.4 m and AUD 49.7 m.

**Conclusion:**

XLRP imposes a substantial burden on quality of life and generates high societal costs relative to direct healthcare costs, consistent with patterns observed in other IRDs. The results highlight the importance of incorporating societal costs into cost-effectiveness analyses when assessing new interventions for individuals with XLRP, including genetic testing and emerging gene therapies.

## Introduction

Retinitis Pigmentosa (RP) is a group of inherited retinal diseases (IRD), causing progressive vision loss often leading to blindness [1]. X-linked retinitis pigmentosa (XLRP) is a severe form of RP and is most commonly caused by mutations in the *Retinitis Pigmentosa GTPase Regulator* (*RPGR)* gene (OMIM#: 312610) [2–4]. XLRP is typically more severe in males, with an earlier onset and more rapid progression to legal blindness than other forms of RP [5]. It is estimated to cause approximately 15% of all RP cases [6], though prevalence among males has been reported from 4% to 20% in different studies [7].

Currently, there are no available treatments for XLRP. Advances in genetic testing for IRDs have provided a way to classify patients by potential genetic causes, including variants in the *RPGR* gene causing XLRP [8, 9] and early-stage trials for gene therapies for XLRP have shown promising results [5, 10–12], including potential improvements in visual outcomes [13].

X-linked retinitis pigmentosa (XLRP) imposes a considerable burden on affected individuals and their families, yet a narrative review has noted a marked absence of research quantifying its impact on quality of life [14]. Although XLRP may impose a greater burden compared to other IRDs [14], there remains a gap in disease-specific data derived from quality of life instruments with utility algorithms, which are essential for economic evaluations. Recent evaluations of gene therapies, including those targeting IRDs, have faced several methodological limitations [15], such as insufficient evidence on broader societal costs—including caregiver burden and use of social services—as well as a lack of utility data specific to the condition [16]. These challenges are especially relevant for XLRP, where productivity losses and informal care needs may represent substantial components of the socioeconomic burden, yet data to support their inclusion in economic evaluations remain limited [17].

Studies on the quality-of-life impacts of IRDs as a whole have found negative impacts, including on mental health [18–21]. However, these studies have not specifically quantified the impacts of XLRP, which has an earlier onset of symptoms and has historically been reported to be more severe than other types of IRD [22]. A systematic review on disease progression in *RPGR*-associated XLRP found median age to legal blindness measured by visual field was 26.4 years [23]. In a study of 113 patients with XLRP caused by *RPGR* variants, the median age of legal blindness was 32 years, much lower than that of those who had RP due to *RHO* variants (77 years) [8]. A recent 2025 study of a sample of 176 patients with XLRP caused by *RPGR* gene mutation showed substantial impacts on unemployment, productivity, mobility and daily activities as the disease progresses. However, while this study provided valuable insights into how XLRP affects patients’ daily lives and well-being, it did not quantify the economic costs associated with these impacts. This gap remains an important limitation for informing economic evaluations.

A 2025 vignette-based study estimated XLRP utilities using UK general population preferences, reporting low values from 0.27 to 0.90 for severe visual impairment and blindness [24]. However, these reflect hypothetical scenarios rather than patient-reported quality of life and may not capture XLRP’s real-world impact or costs. No studies have yet combined patient-derived utility data with cost estimates from a clinical cohort of patients with XLRP, underscoring the need for further research to support economic evaluations of new treatments.

Multiple studies on the costs of IRDs in different countries have found high costs, including societal costs [25–28]. Studies on RP have also shown lower rates of employment compared to age-paired controls without the disease [29]. However, these studies may not reflect the costs and burden of XLRP specifically.

Review of the literature indicates that there is currently no estimate of health-related quality of life (HRQoL) and the associated health utilities from patients with XLRP using a recognised preference-based instrument, nor a comprehensive study of the societal and economic costs of the disease. In this paper, we use primary data from the Economic and Psychosocial Impact of Caring for Families Affected by Visual Impairment (EPIC-Vision) study [27]. This study recruited a clinically ascertained cohort of patients diagnosed with XLRP caused by RPGR variants to estimate the HRQoL impacts and utility values associated with the disease. A microsimulation model was used to estimate the lifetime health and societal costs of XLRP. To our knowledge, this is the first study to combine patient-reported utility values with comprehensive health and societal cost estimates from a clinically diagnosed cohort of patients with XLRP caused by variants in RPGR.

## Methods

A cohort of IRD patients and their carers was recruited from statewide services in New South Wales, Australia. All adult patients with a clinical diagnosis of IRD and their carers were invited to participate at two locations: The Children’s Hospital at Westmead, Sydney Children’s Hospitals Network and Sydney Eye Hospital and Save Sight Institute [20]. Children with IRD were invited to participate via their carers, with carers responding with a proxy survey on behalf of their child. XLRP patients with variants in *RPGR* were selected from this cohort. All participants and their carers were administered the Economic and Psychosocial Impact of Caring for Families Affected by Visual Impairment (EPIC-Vision) questionnaire [27], a tailored questionnaire covering domains of impact of the disease.

### Ethics approval

The EPIC-Vision study was approved by the Sydney Children’s Hospital Network Human Research Ethics Committee (HREC/18/SCHN/292).

#### Health-related quality of life impacts

HRQoL for adults was measured using the AQoL-8D, a validated HRQoL instrument to estimate health utilities [30]. The AQoL-8D measures quality of life across 8 domains: independent living, sense, pain, mental health, happiness, self-worth, coping and relationships. The score from each domain is then used to estimate a total utility value using the algorithm associated with the instrument [31]. The AQoL-8D was preferred as the HRQoL instrument due to the specific dimensions relevant for XLRP, including vision, social, relationships, mental health and other psychosocial questions [20]. For children, the health utilities index (HUI) 2 and 3 were used to assess quality of life.

To compare utility values of patients with XLRP to the general population, one-sample t-tests were performed against population norms of the AQoL-8D [31], with a significance level set at 5%. Population norms were adjusted for age and gender to reflect the composition of the cohort with XLRP.

#### Microsimulation model and costs

To estimate the costs of XLRP, a microsimulation model was used [27], with a combination of primary data collected from EPIC-Vision and administrative health data. Microsimulation enables the modelling of disease progression and associated costs at the individual level, supporting more precise estimates of costs and impacts over time.

#### Healthcare costs

Healthcare costs for XLRP patients are a combination of self-reported hospital use data via the EPIC-Vision questionnaire and administratively linked Medicare data through the Medicare Benefits Schedule (MBS) and the Pharmaceutical Benefits Scheme (PBS). MBS data contains the use and prices of all medical services in Australia funded by Medicare. PBS data contains prices and costs of all prescriptions used in Australia for medicines funded under the scheme. A full list of health costs used in the study is included in Appendix 1.

#### Societal costs

Societal costs reflect all costs not attributable to the health system. These are estimated by source of cost and payer: patients and carers, federal government, state government and others. Patients and carers provide information on receipt of government payment services and supports, purchase of aids and appliances, receipt of income and other support payments and use of the National Disability Insurance Scheme (NDIS), Australia’s national insurance scheme for those with a disability. Some costs, such as the use of aids and appliances, spanned multiple years, with all amounts converted to annual costs. The full list of societal costs is provided in Appendix 1.

#### Income and tax losses

To estimate the income and tax effects of IRDs, we derive data from the Static Income Model (STINMOD) [32]. STINMOD is a tax-benefit model that provides a representative sample of Australian households. Patients with XLRP and their carers are matched to the general population using gender, state, education and age, with 1000 simulations run. The difference between the XLRP cohort and the average from the simulations reflects the income and tax effects of XLRP.

#### Inflation

All prices are updated to June 2024 prices using inflation specific to the type of cost. Further details are provided in Appendix 1.

#### Annual and lifetime costs

All costs are converted to annual amounts and patients are grouped by age into categories:0-17, 18-29, 30-39, 40-49, 50-59, 60-69, 70-84. Average annual costs are then estimated for each age group. Average annual costs are projected to average life expectancy (84) in Australia [33] to produce an estimated lifetime cost of XLRP. As no XLRP patients aged over 60 participated, data on costs from patients with RP in the EPIC-Vision study were used to estimate XLRP costs for 60–69 and 70–84 year age groups [27]. The costs of RP patients were adjusted for the ratio of XLRP to RP costs in other age groups to estimate XLRP costs in these age groups.

#### Prevalence and national estimates

To estimate the national annual costs of XLRP, prevalence estimates for XLRP were obtained from international literature [7, 34, 35]. This produced a lower bound of 3.4 males per 100,000 and an upper bound of 4.4 males per 100,000. These were combined with Australian population estimates [36] to produce estimates of total annual national costs of XLRP in Australia.

All statistical analyses were performed in SAS 9.4.

## Results

Descriptive statistics of the XLRP cohort are shown in Table 1. 9 adult patients with XLRP responded to the survey and 4 carers filled out surveys on behalf of patients they cared for. The mean age of the adult cohort was 38.4 years and 12.0 years for children, while the mean age of carers was 48.8. All patients in the cohort were male. For the child cohort, the average age of symptom onset was 5.5 years and 7.4 years for the adult cohort.

**Table 1:** Descriptive statistics of the XLRP patient cohort and their carers in EPIC-Vision.

Characteristic	Adult Cohort	Child Cohort
Age (Average, years)		
Adult cohort	38.4 (11.5)	12.0 (1.7)
Carers	57	48.8
Gender		
Male	9 (100%)	3 (100%)
Symptom onset		
Age (Average, years)	7.4 (6.3)	5.5 (4.3)
Visual acuity		
Normal to moderate visual impairment (better than 6/60)	6 (66.7%)	3 (100%)
Severe and profound visual impairment (Worse than 6/60)	2 (22.2%)	
Missing	1 (11.1%)	0 (0%)
Visual Field		
Reduced visual field or decreased peripheral vision		
Yes	8 (88.9%)	2 (66.7%)
No	0 (0%)	1 (33.3%)
Unknown	1 (11.1%)	
Difficulty seeing at night		
Yes	9 (100%)	2 (66.7%)
No	0 (0%)	2 (33.3%)

### HRQoL (utility values)

**Fig. 1:**
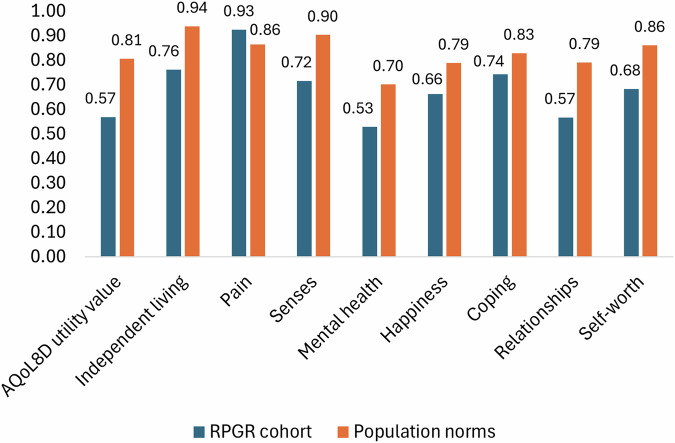
AQoL-8D dimension scores and utility values for adult patients with XLRP and population norms.

AQoL-8D utility values for adult patients with XLRP were 0.57 (SD 0.14, 95% CI 0.46-0.68), compared to 0.81 in age and gender adjusted population norms (Fig. 1). T-tests showed AQoL-8D utility values were significantly lower (*p* < 0.05) for people with XLRP (*p* = 0.001), with scores on the independent living, senses, mental health, relationships and self-worth domains significantly lower than population norms; 0.76 vs 0.94 (*p* = 0.008), 0.72 vs 0.90 (*p* = 0.002), 0.53 vs 0.70 (*p* = 0.005), 0.57 vs 0.79 (*p *= 0.001) and 0.68 vs 0.86 respectively (*p* = 0.002).

**Fig. 2:**
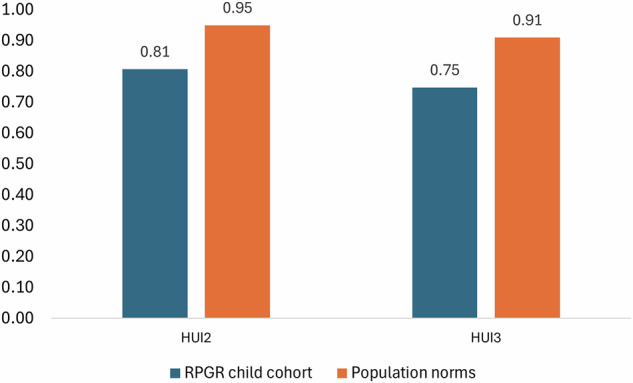
HUI2 and HUI3 utility values for child patients with XLRP and population norms.

For children with XLRP, HUI2 and HUI3 values were 0.81 (SD = 0.17) and 0.75 (SD – 0.24), compared to population norms of 0.95 and 0.91, respectively (Fig. 2). Given the small sample size (n = 3), formal hypothesis testing was not conducted.

### Costs

Lifetime annual costs per XLRP patient were AUD 7.0 m (Table 2). The highest annual costs were for 40–49-year-olds and 50-59-year-olds. This was due to the loss of income to patients and the corresponding lower tax receipts to the government.

**Table 2:** Annual and total costs of XLRP by age group, category and payer.

Age group	Annual costs ($)	Cumulative cost ($)
0 to 29	91,191	27,35,739
30–39	39,261	31,28,349
40–49	1,41,954	45,47,888
50–59	1,47,643	60,24,315
60+	40,677	70,41,248

Over the lifetime, combined lost income for patients, carers and their spouses totalled approximately AUD 3.5 m (Table 3). The combined Commonwealth and State NDIS costs were estimated at AUD 525 139, while other social spending on patients and carers was over AUD 750 000. As Table 3 shows, the majority of lifetime costs were societal, with health costs comprising 22% of costs.

**Table 3:** Lifetime costs of XLRP by category and payer.

Commonwealth government	$
Health	6,40,585
Social spending excl National Disability Insurance Scheme (NDIS) - patient	3,81,792
NDIS	2,96,776
Social spending - carer	2,46,832
Tax receipts - patient	9,58,921
Tax receipts - carer/spouse	3,52,530
**Total**	**28,77,436**
**State Government**	
Health	6,08,485
Social spending - patient	1,13,782
NDIS	2,14,907
**Total**	**9,37,174**
**Individual/household**	
Health	2,37,673
Other including aids/modifications	32,770
Lost income - patient	25,59,598
Lost income - spouse/carer	9,01,550
**Total**	**37,31,592**
**Total**	**75,46,201**
**Total cost (after adjustment)**^a^	**70,41,248**

^a^The estimated total costs will diverge from the sum of its components because of adjustment to avoid double counting as welfare payments replace lost income.

### Sensitivity analysis—outlier costs

Primary data revealed substantial health costs in outliers unrelated to the treatment of XLRP. As a sensitivity analysis, these costs for treatments for chronic and acute conditions unrelated to XLRP were removed from the analysis. If these are removed from the analysis, total costs fall to $6.2 m.

### National cost estimates

Using 2024 Australian population estimates, the total national annual costs of XLRP in Australia are estimated to be between AUD 38.4 m and AUD 49.7 m per annum.

## Discussion

Adult patients with XLRP have substantially lower quality of life measured through health utilities (0.57) compared to age and gender adjusted population norms of 0.81. On individual domains of the AQoL-8D, patients had lower scores in independent living, senses, mental health, coping, relationships and self-worth, reflecting how the disease impacts all aspects of life.

The lifetime cost per patient with XLRP was AUD 7.0 million. Like other IRDs, societal costs comprised the greatest share (79%) of costs. The lifetime lost income costs of AUD 2.6 million for patients and AUD 901,550 for carers reflect the impact XLRP has on the ability to work. These findings are consistent with findings for other IRDs; however, the lost income for those with XLRP appears to be greater than the overall EPIC-Vision cohort encompassing all IRDs [27], possibly reflecting an earlier onset and thus a larger impact on patients’ ability to remain employed.

XLRP patients used a variety of government services, including health, welfare and social supports, including the NDIS. Lifetime NDIS costs were approximately AUD 512000, reflecting the needs of many IRD patients outside the health system, while social support payments were lower at approximately AUD 901,550 for both patients and carers.

While the economic and societal burden of XLRP is substantial, advances in genetic testing and gene therapies provide real promise to patients. Early-stage trials for gene therapies for XLRP have demonstrated some improvements in visual fields [37]. However, public or other funding for these will require evidence on the potential benefits and costs. Previous cost-effectiveness studies on gene therapies have suffered from a lack of data on the burden of the disease [16], including disease-specific quality of life, utility impacts and costs.

While other studies on IRDs as a whole have estimated the QoL impacts [20, 27] and costs, they may not be reflective of the burden of XLRP [14]. In this paper, we address this gap using data from a clinically ascertained cohort of XLRP patients. We estimated the quality-of-life impacts of XLRP, showing substantial negative impacts, significantly below population norms and just lower than previous results (0.58 vs 0.57) on quality of life and utility for patients with IRDs [20]. A previous 2025 vignette-based study estimated hypothetical utilities for XLRP [24]. Our study provides patient-reported data that more directly reflects disease burden. While vignette-based methods have been used when patient samples are difficult to access, as is common in rare diseases, they have inherent limitations: the utilities reflect hypothetical preferences rather than lived experiences [38]. This limits their ability to capture the full scope of impacts or use standardise measures [38]. Where patient data is available, as in the clinical cohort in this study, patient-reported utilities from recognised instruments are preferable as they directly reflect real-world disease burden. Patient-derived utility data is essential for informing future evaluations of gene therapies, particularly as recent studies have cautioned that current economic models often underestimate the value of treatments in rare conditions like XLRP due to a lack of primary data [17].

The costs of XLRP estimated in this paper demonstrate the significant burden associated with the disease, with a much higher portion of societal (79%) compared to health (21%) costs. This demonstrates why future cost-effectiveness analyses should consider societal costs when estimating the costs of XLRP. As highlighted in recent work on gene therapy for XLRP, the absence of societal cost data poses a risk of undervaluing treatments, particularly given their potential to improve independence, workforce participation and reduce caregiving demands [17]. The high societal burden of costs, particularly due to lower workforce participation for both patients and carers, also demonstrates where targeted policy could assist in improving outcomes for families with XLRP.

This paper shows that the lifetime costs of XLRP are higher than those of other IRDs in Australia, estimated at AUD 7.0 m and AUD 5.2 m, respectively [27]. This difference is largely explained by higher lost incomes for patients and the disease being more likely to affect males, consistent with other studies on the cost of IRDs. If outliers are removed from XLRP, the differences in costs are reduced, though the costs of XLRP still remain higher than those of other IRDs in the EPIC-Vision study.

### Strengths and limitations of paper

A key strength of this paper is the use of data from a clinically diagnosed cohort of patients with XLRP, providing necessary data for cost-effectiveness analysis of any interventions, including genetic testing and targeted therapies. The small sample size increases uncertainty in our estimates and limits the ability to stratify results by disease severity. As such, the costs and utility values reported should be considered indicative.

## Conclusion

Patients with XLRP have significantly lower quality of life compared to the general population. Lifetime costs of $7.0 m per patient and national annual costs of between $38.4 m and $49.7 m reveal a high-cost burden, with 79% comprised of societal costs. The high societal costs demonstrate the need for their inclusion in cost-effectiveness analysis for interventions for XLRP, including genetic testing and gene therapies.

## Summary

### What is known about this topic

- XLRP, like other IRDs, poses a substantial burden on patients, with an earlier onset of symptoms than other IRDs.
- Gene therapies for XLRP are showing some promise in trials.

### What this study adds

- XLRP imposes substantial costs on patients, families and governments, with societal costs forming a much larger proportion of all costs than health costs.
- XLRP substantially reduces the quality of life for patients.
- This study provides health utility data and comprehensive cost data for economic evaluations of interventions for XLRP, including gene therapies.

## Supplementary information

Appendix 1: Data sources and methods for societal and health costs of XLRP

## Data Availability

Under the terms of our ethics agreement, information obtained in connection with this research project must remain confidential and the study data cannot be shared beyond the research team.

## References

[1] Ferrari S, Di Iorio E, Barbaro V, Ponzin D, Sorrentino FS, Parmeggiani F. Retinitis pigmentosa: genes and disease mechanisms. Curr Genomics. 2011;12:238–49. 22131869 10.2174/138920211795860107 PMC3131731

[2] Beltran WA, Cideciyan AV, Lewin AS, Hauswirth WW, Jacobson SG, Aguirre GD. Gene augmentation for X-linked retinitis pigmentosa caused by mutations in RPGR. Cold Spring Harb Perspect Med. 2014;5:a017392. 25301933 10.1101/cshperspect.a017392 PMC4315918

[3] Zhang Q, Giacalone JC, Searby C, Stone EM, Tucker BA, Sheffield VC. Disruption of RPGR protein interaction network is the common feature of RPGR missense variations that cause XLRP. Proc Natl Acad Sci USA. 2019;116:1353–60. 30622176 10.1073/pnas.1817639116 PMC6347721

[4] Zada M, Cornish EE, Fraser CL, Jamieson RV, Grigg JR. Natural history and clinical biomarkers of progression in X-linked retinitis pigmentosa: a systematic review. Acta Ophthalmol. 2021;99:499–510. 33258268 10.1111/aos.14662

[5] Martinez-Fernandez De La Camara C, Nanda A, Salvetti AP, Fischer MD, MacLaren RE. Gene therapy for the treatment of X-linked retinitis pigmentosa. Expert Opin Orphan Drugs. 2018;6:167–77. 30057863 10.1080/21678707.2018.1444476 PMC6059358

[6] Branham K, Othman M, Brumm M, Karoukis AJ, Atmaca-Sonmez P, Yashar BM, et al. Mutations in RPGR and RP2 account for 15% of males with simplex retinal degenerative disease. Invest Ophthalmol Vis Sci. 2012;53:8232–7. 23150612 10.1167/iovs.12-11025 PMC3522443

[7] Vinikoor-Imler LC, Simpson C, Narayanan D, Abbasi S, Lally C. Prevalence of RPGR-mutated X-linked retinitis pigmentosa among males. Ophthalmic Genet. 2022;43:581–8. 36004681 10.1080/13816810.2022.2109686

[8] Sandberg MA, Rosner B, Weigel-DiFranco C, Dryja TP, Berson EL. Disease course of patients with X-linked retinitis pigmentosa due to RPGR gene mutations. Investig Ophthalmol Vis Sci. 2007;48:1298–304. 17325176 10.1167/iovs.06-0971

[9] Li J, Tang J, Feng Y, Xu M, Chen R, Zou X, et al. Improved diagnosis of inherited retinal dystrophies by high-fidelity PCR of ORF15 followed by next-generation sequencing. J Mol Diagnost. 2016;18:817–24. 10.1016/j.jmoldx.2016.06.007 27620828

[10] von Krusenstiern L, Liu J, Liao E, Gow JA, Chen G, Ong T, et al. Changes in retinal sensitivity associated with cotoretigene toliparvovec in X-linked retinitis pigmentosa with RPGR gene variations. JAMA Ophthalmol. 2023;141:275–83. 36757689 10.1001/jamaophthalmol.2022.6254 PMC9912164

[11] Beltran WA, Cideciyan AV, Boye SE, Ye GJ, Iwabe S, Dufour VL, et al. Optimization of retinal gene therapy for X-linked retinitis pigmentosa due to RPGR mutations. Mol Ther. 2017;25:1866–80. 28566226 10.1016/j.ymthe.2017.05.004 PMC5542804

[12] Mansouri V. X-linked retinitis pigmentosa gene therapy: preclinical aspects. Ophthalmol Ther. 2023;12:7–34. 36346573 10.1007/s40123-022-00602-y PMC9641696

[13] Sengupta S. Perspectives on evolving gene therapy for X-linked retinitis pigmentosa. JAMA Ophthalmol. 2023;141:283–4. 36757711 10.1001/jamaophthalmol.2022.6436

[14] Chivers M, Li N, Pan F, Wieffer H, Slowik R, Leartsakulpanitch J. The burden of X-linked retinitis pigmentosa on patients and society: a narrative literature review. Clinicoecon Outcomes Res. 2021;13:565–72. 34188501 10.2147/CEOR.S297287 PMC8236258

[15] Huygens SA, Versteegh MM, Vegter S, Schouten LJ, Kanters TA. Methodological challenges in the economic evaluation of a gene therapy for RPE65-mediated inherited retinal disease: the value of vision. Pharmacoeconomics. 2021;39:383–97. 33604870 10.1007/s40273-021-01003-y PMC8009797

[16] Pochopień M, Paterak E, Clay E, Janik J, Aballea S, Biernikiewicz M, et al. An overview of health technology assessments of gene therapies with a focus on cost-effectiveness models. J Mark Access Health Policy. 2021;9:2002006. 34790341 10.1080/20016689.2021.2002006 PMC8592603

[17] Hitch J, Denee T, Brassel S, Lee J, Michaelides M, Petersen J, et al. Challenges in value assessment for one-time gene therapies for inherited retinal diseases: are we turning a blind eye? Value Health. 2025;28:116–24. 39384069 10.1016/j.jval.2024.08.009

[18] Chaumet-Riffaud AE, Chaumet-Riffaud P, Cariou A, Devisme C, Audo I, Sahel JA, et al. Impact of retinitis pigmentosa on quality of life, mental health, and employment among young adults. Am J Ophthalmol. 2017;177:169–74. 28237413 10.1016/j.ajo.2017.02.016

[19] Altinbay D, Taskin I. Evaluation of vision-related quality of life in retinitis pigmentosa patients with low vision. Jpn J Ophthalmol. 2021;65:777–85. 34606034 10.1007/s10384-021-00875-z

[20] Schofield D, Kraindler J, Tan O, Shrestha R, Jelovic D, West S, et al. Patient-reported health-related quality of life in individuals with inherited retinal diseases. Ophthalmol Sci. 2022;2:100106. 36246188 10.1016/j.xops.2021.100106 PMC9560564

[21] McGuinness MB, Ayton LN, Schofield D, Britten-Jones AC, Chen FK, Grigg JR, et al. EQ-5D-5L health utility scores in Australian adults with inherited retinal diseases: a cross-sectional survey. Acta Ophthalmol. 2024;102:e736–e45. 38226448 10.1111/aos.16634

[22] Lam BL, Scholl HPN, Doub D, Sperling M, Hashim M, Li N. A systematic literature review of disease progression reported in RPGR -associated x-linked retinitis pigmentosa. Retina. 2024;44:1–9. 37683184 10.1097/IAE.0000000000003920

[23] Lam BL, Scholl HPN, Doub D, Sperling M, Hashim M, Li N A Systematic literature review of disease progression reported in RPGR-associated X-linked retinitis pigmentosa. Retina. 2024;44:1–9. 10.1097/IAE.0000000000003920 37683184

[24] Matza LS, Li N, Stewart KD, Hashim M, Denee T, Pan F, et al. Health state utilities associated with X-linked retinitis pigmentosa (XLRP). Eur J Health Econ 2025;26:1263–73. 10.1007/s10198-025-01761-y PMC12431885 40095340

[25] Galvin O, Chi G, Brady L, Hippert C, Del Valle Rubido M, Daly A, et al. The impact of inherited retinal diseases in the Republic of Ireland (ROI) and the United Kingdom (UK) from a cost-of-illness perspective. Clin Ophthalmol. 2020;14:707–19. 32184557 10.2147/OPTH.S241928 PMC7062501

[26] Gong J, Cheung S, Fasso-Opie A, Galvin O, Moniz LS, Earle D, et al. The impact of inherited retinal diseases in the United States of America (US) and Canada from a cost-of-illness perspective. Clin Ophthalmol. 2021;15:2855–66. 34234408 10.2147/OPTH.S313719 PMC8257071

[27] Schofield D, Kraindler J, Tan O, Shrestha RN, West S, Hart N, et al. The health care and societal costs of inherited retinal diseases in Australia: a microsimulation modelling study. Med J Aust. 2023;219:70–6. 37301731 10.5694/mja2.51997 PMC10952471

[28] Chay J, Tang RWC, Tan TE, Chan CM, Mathur R, Lee BJH, et al. The economic burden of inherited retinal disease in Singapore: a prevalence-based cost-of-illness study. Eye. 2023;37:3827–33. 37301937 10.1038/s41433-023-02624-7 PMC10698171

[29] Cross N, van Steen C, Zegaoui Y, Satherley A, Angelillo L. Retinitis pigmentosa: burden of disease and current unmet needs. Clin Ophthalmol. 2022;16:1993–2010. 35757022 10.2147/OPTH.S365486 PMC9232096

[30] Richardson J, Iezzi A, Khan MA, Maxwell A. Validity and reliability of the assessment of Quality of Life (AQoL)-8D multi-attribute utility instrument. Patient. 2014;7:85–96. 24271592 10.1007/s40271-013-0036-x PMC3929769

[31] Maxwell A, Özmen M, Iezzi A, Richardson J. Deriving population norms for the AQoL-6D and AQoL-8D multi-attribute utility instruments from web-based data. Qual Life Res. 2016;25:3209–19. 27344318 10.1007/s11136-016-1337-z

[32] Lambert S, Percival, R, Schofield, D, Paul, S. An introduction to STINMOD: a static microsimulation model. (STINMOD technical paper; No. 1). National Centre for Social and Economic Modelling (NATSEM). 1994.

[33] Australian Bureau of Statistics. Life tables, 2019–2021 [Available from: https://www.abs.gov.au/statistics/people/population/life-tables/2019-2021.

[34] Bunker CH, Berson EL, Bromley WC, Hayes RP, Roderick TH. Prevalence of retinitis pigmentosa in Maine. Am J Ophthalmol. 1984;97:357–65. 6702974 10.1016/0002-9394(84)90636-6

[35] Prokisch H, Hartig M, Hellinger R, Meitinger T, Rosenberg T. A population-based epidemiological and genetic study of X-linked retinitis pigmentosa. Investig Ophthalmol Vis Sci. 2007;48:4012–8. 17724181 10.1167/iovs.07-0071

[36] Australian Bureau of Statistics. National, state and territory population 2024 [Available from: https://www.abs.gov.au/statistics/people/population/national-state-and-territory-population/jun-2024.

[37] Martínez-Fernández de la Cámara C, Cehajic-Kapetanovic J, MacLaren RE. Emerging gene therapy products for RPGR-associated X-linked retinitis pigmentosa. Expert Opin Emerg Drugs. 2022;27:431–43. 36562395 10.1080/14728214.2022.2152003

[38] Matza LS, Stewart KD, Lloyd AJ, Rowen D, Brazier JE. Vignette-based utilities: usefulness, limitations, and methodological recommendations. Value Health. 2021;24:812–21. 34119079 10.1016/j.jval.2020.12.017

