# Supplementary material for: The quality-of-life impacts and economic burden of X-linked retinitis pigmentosa caused by variants in RPGR
Source: Eye (Lond). 2026 May 21;40(9):1302–7. doi: 10.1038/s41433-026-04532-y (PMC13269770; doi:10.1038/s41433-026-04532-y)
Supplement: Supplementary file 1 — Appendix 1: Data sources and methods for societal and health costs of XLRP [file 41433_2026_4532_MOESM1_ESM.docx]

Appendix 1: Data sources and methods for societal and health costs of XLRP (27)

| **Items** | **Cost and source** | **Perspective** |
| --- | --- | --- |
| **Health** | | |
| Out of hospital Medicare funded services | Medicare Benefits Schedule (MBS) – item number, schedule fee and out of pocket cost. Data received is from year of interview and all four years prior, with annual average calculated for model.  EPIC-Vision* | Federal government and individual |
| Prescription medicine | Pharmaceutical Benefits Scheme (PBS) item number plus out of pocket cost  Data received is from year of interview and all four years prior, with annual average calculated for model.  EPIC-Vision* | Federal government and individual |
| Non-prescription medicines and supplements | EPIC-Vision reported costs | Individual |
| Public inpatient | Hospital utilisation and average cost of NSW bed day | Federal and state government |
| Private inpatient | Hospital utilisation and average cost of bed day (private hospital), MBS item number | Federal government, private insurance and individual |
| Emergency department | Emergency attendance and average emergency attendance cost in NSW | Federal and state government |
| Transport, accommodation and food during hospital care | EPIC-Vision | Individual |
| Allied and other health services, excluding MBS, such as orthoptics | EPIC-Vision, imputed where utilisation was reported without costs | Federal government and individual |
| **Income and employment** | | |
| Employment status | EPIC-Vision | Individual |
| Income loss | EPIC-Vision and STINMOD | Individual |
| Tax receipts | EPIC-Vision and STINMOD | Federal government |
| **Societal** | | |
| **National Disability Insurance Scheme (NDIS) and social support** | | |
| Specialist Disability Accommodation | EPIC-Vision and National Disability Insurance Agency (NDIA) price guide | Federal government |
| Supported independent living | EPIC-Vision and National Disability Insurance Agency (NDIA) price guide | Federal government |
| Respite care | EPIC-Vision and NDIA price guide | Federal government |
| Rental support | EPIC-Vision and current rent subsidies | Federal government |
| Public Housing | EPIC-Vision | State government |
| NDIS package | EPIC-Vision – Current NDIS package amount | Federal and state governments – funding split according to 2021-22 federal budget. |
| **Income and welfare support** | | |
| Income support including job keeper, aged and disability pensions | EPIC-Vision and Department of Social Services Social Security Guide | Federal government |
| Primary and secondary school support | EPIC-Vision and Department of Education, Skills and Education funding guides | Federal and state government |
| Early intervention class | EPIC-Vision and NDIA price guide | Federal and state government |
| Preschool | EPIC-Vision and Department of Education, Skills and Education funding guides | Federal and state government |
| **Aids and modifications** | | |
| Household aids and modifications | EPIC-Vision | Federal government and individual |
| Education aids and modifications | EPIC-Vision | Federal government and individual |
| Transport aids and modifications | EPIC-Vision | Federal government and individual |
| Guide Dogs | EPIC-Vision plus cost of training | Federal government and individual |

Wages were adjusted for inflation using the Wage Prices Index. Health costs were adjusted using annual health inflation from the AIHW, as well as index factors for the Medicare Benefits Schedule. NDIS costs were adjusted using NDIS inflation. Other costs were adjusted using the Consumer Price Index (CPI).

*Where a patient did not consent to data linkage, costs were estimated based on patient responses in EPIC-Vision as participants were asked specific questions about use of all types of health services, including both Medicare and non-Medicare funded services.
